# High Self-compassion Despite Elevated Stress and Reduced Quality of Life in Women with Lipoedema

**DOI:** 10.2340/actadv.v106.adv-2025-0236

**Published:** 2026-06-11

**Authors:** Johan Dahlberg, Elisabet Nylander, Jens Boman, Alexander Shayesteh

**Affiliations:** 1 Department of Public Health and Clinical Medicine, Dermatology and Venereology, Umeå University, Umeå, Sweden; 2 Department of Clinical Sciences, Professional Development, Umeå University, Umeå, Sweden

Lipoedema is a chronic disorder characterised by painful accumulation of adipose tissue, almost exclusively affecting women (1, 2). Clinically, lipoedema presents as bilateral, symmetrical adipose tissue hypertrophy, typically affecting hips, thighs, lower legs and occasionally the arms (1). Lipoedema is classified according to anatomical distribution and stage of progression, commonly described in four stages (1, 2). In addition to physical symptoms, lipoedema is associated with reduced quality of life, impaired psychological well-being and increased prevalence of mental health conditions such as anxiety and depression (2–7).

To the best of our knowledge, there is no study investigating the prevalence of self-compassion, perceived stress, generalised anxiety disorder, perfectionism and impostor phenomenon among women with lipoedema. Understanding the presence and potential interaction of these factors could enable a better understanding of psychological resilience and vulnerability and facilitate areas for future interventions.

## MATERIALS AND METHODS

### Participants

Twenty-six women with lipoedema were included. All participants were over 18 years of age, had been diagnosed with lipoedema based on the criteria from Wold et al*.* (8) and Halk et al*.* (9) and confirmed by a dermatologist with clinical experience in lipoedema (JD).

### Psychometric instruments and questionnaires

A set of psychometric instruments was used to assess symptoms and traits related to stress (PSS-4), anxiety (GAD-2), self-compassion (SCS-SF), perfectionism (CPQ-6) and the impostor phenomenon (CIPS) (Appendix S1). The Swedish versions of the questionnaires have previously been used by us to investigate psychological profiles in clinical populations, including individuals with primary hyperhidrosis (10) and localized provoked vulvodynia (11). In addition, health-related quality of life was examined using RAND-36, a validated questionnaire with a physical component summary (PCS) and a mental component summary (MCS) as well as 8 subscales, each with a range of 0 to 100 points (12) (Appendix S1).

### Statistical analysis

As prior effect size estimates were unavailable and the number of eligible patients with lipoedema was limited, no formal sample size or power calculation was conducted. Instead, all individuals meeting the inclusion criteria during the recruitment period were invited to participate. Due to the small number of participants with stage 1 lipoedema, they were grouped with participants with stage 2 lipoedema to enable meaningful statistical comparison. Descriptive statistics were used to summarise participant characteristics. Between-group comparisons for the RAND-36 were calculated using independent samples *t*-test when data were normally distributed; otherwise, the Mann–Whitney *U* test was utilised. Significance was set at *p*<0.05, and all statistical analyses were conducted using IBM SPSS Statistics (version 30.0.0.0).

## RESULTS

A total of 18 women with lipoedema participated. Background characteristics are presented in Table I.

**Table I.** Characteristics of participants

	All (*n*=18)	Stage 1 and 2 (*n*=10)	Stage 3 (*n*=8)
Age, median (IQR)	54 (40.8–64.3)	54 (44.8–66.5)	54 (37.8–59.8)
Lipoedema stage, *n* (%)			
1	2 (11.1)	na	na
2	8 (44.4)	na	na
3	8 (44.4)	na	na
PSS-4, mean±SD	6.7±2.1	7.0±2.4	6.4±1.9
GAD-2, mean±SD	1.3±1.6	1.5±1.8	1.1±1.4
SCS-SF, mean±SD	3.4±0.7	3.3±0.6	3.5±0.7
CPQ-6, mean±SD	14.6±2.8	15.1±2.1	13.9±3.5
CIPS, mean±SD	52.1±17.8	56.8±19.8	46.1±14.0

CIPS: Clance Impostor Phenomenon Scale; CPQ-6: Clinical Perfectionism Questionnaire-6; GAD-2: Generalised Anxiety Disorder Scale-2; IQR: interquartile range; n: number; na: not applicable; PSS-4: Perceived Stress Scale-4; SCS-SF: Self-Compassion Scale Short Form; SD: standard deviation.

### Stress, anxiety, self-compassion, perfectionism and impostor phenomenon

Signs of elevated stress were found to be prevalent in one third of the women. Regarding anxiety, 16.7% of the participants reported an elevated level. In SCS-SF, 88.9% of participants reported a moderate or high level of self-compassion, among whom 9 reported a high level of self-compassion. A total of 61.1% rated their perfectionism as moderate, 33.3% as difficult, and none as severe. In CIPS, 27.8% had a score of 62 or higher, indicating elevated levels of imposter feelings. Based on the CIPS classification, 11.1% of the participants experienced intense feelings of imposter phenomenon (IP), 16.7% frequent feelings, 38.9% moderate feelings and 33.3% few feelings of IP. There were no significant differences between the lipoedema subgroups stage 1 and 2 and the lipoedema stage 3 regarding PSS-4, GAD-2, SCS-SF, CPQ-6 and CIPS.

### RAND-36

The mean results from the RAND-36 showed that emotional well-being (65.1±13.1) had the highest score, followed by physical function (50.6±22.3) and social functioning (49.3±23.7). The remaining subscale mean scores were as follows: general health (39.4±19.0), health change (37.5±33.5), energy/fatigue (36.4±16.8), pain (34.2±16.9), role emotional (33.3±39.6) and role physical (22.2±34.2). The PCS was 31.3±9.32, and the MCS was 38.1±9.56. Scores comparing the lipoedema stage groups are found in Table II.

**Table II.** RAND-36 among women with lipoedema, stratified by lipoedema stage

	Stage 1 and 2 Mean±SD	Stage 3 Mean±SD	*p*-value
Physical function	57.0±19.6	42.5±24.1	0.177^a^
Role physical	22.5±36.2	21.9±33.9	0.829^b^
Role emotional	20.0±28.1	50.0±47.1	0.203^b^
Energy/fatigue	37.5±15.5	35.0±19.3	0.764^a^
Emotional well-being	64.0±15.6	66.5±10.0	0.700^a^
Social functioning	43.8±22.2	56.3±25.0	0.278^a^
Pain	38.3±20.3	29.1±10.5	0.264^a^
General health	41.5±12.7	36.9±25.6	0.651^a^
Health change	35.0±39.4	40.6±26.5	0.573^b^
PCS	34.5±7.61	27.3±10.2	0.107^a^
MCS	34.3±9.11	42.9±8.27	0.055^a^

^a^Independent *t*-test for statistical test of difference. ^b^Mann–Whitney *U* test for statistical test of difference. Significance level *p*<0.05.

MCS: mental component summary; PCS: physical component summary; SD: standard deviation.

## DISCUSSION

To our knowledge, this is the first study comprehensively examining impostor phenomenon, perfectionism, self-compassion, stress and signs of anxiety among women with lipoedema. Our findings show that elevated stress levels, high self-compassion and reduced self-reported quality of life are commonly reported by women with lipoedema. The low scores in the RAND-36 role-emotional and role-physical functioning domains highlight limitations in everyday physical activities and reduced mental health among participants. One third of participants also reported elevated stress levels and an elevated degree of pain in RAND-36, consistent with previous findings of impaired mental health in patients with lipoedema (2–7). Despite these results, we could not find increased levels of perfectionism, high levels of anxiety or frequent feelings of impostor phenomenon in women with lipoedema. In contrast, half of the participants reported high levels of self-compassion, which might be due to the relatively high emotional well-being scores regardless of the psychosocial limitations described.

Quality of life in lipoedema, as measured by RAND-36, has been investigated in several studies (6, 7). While our results confirm reduced RAND-36 scores among women with lipoedema, we also report considerably lower scores than previously described (6, 7). We interpret these findings to suggest that the psychological and physical burden of lipoedema may be greater than currently recognised, and that additional factors, such as comorbid mental distress or delayed diagnosis, may also contribute to the diminished quality of life. Further studies are warranted to clarify the factors contributing to impaired quality of life and to inform effective management approaches in lipoedema.

Self-compassion has been strongly associated with health-promoting behaviours, improved treatment adherence and as a negative predictor of pain intensity (13–15). This indicates that high self-compassion could act as a protective factor for both pain management and psychological health in women with lipoedema. However, further research is needed to confirm our findings.

### Strengths and limitations

A major strength of this study is that the diagnosis of lipoedema was clinically validated according to established criteria, ensuring diagnostic accuracy. In addition, the use of multiple patient-reported outcome measures, including the RAND-36, provided a multidimensional view of health-related quality of life in an understudied population. This comprehensive approach contributes valuable data to the growing body of evidence on the impact of lipoedema.

Nonetheless, several limitations should be considered. The sample size was limited, reducing statistical power and potentially affecting the generalisability of the findings. However, due to the challenges of studying lipoedema, an under-recognised condition without consensus regarding its prevalence or objective methods for diagnosis and often overlapping with other disorders, recruiting a large, well-characterised sample would have been particularly difficult to achieve. Despite this, our findings could provide valuable insights into the psychosocial and clinical profile of affected women.

### Conclusion

This study indicates that lipoedema is associated with reduced quality of life, as reflected in lower RAND-36 scores and elevated stress levels. At the same time, women in this cohort reported high levels of self-compassion, which may represent an important psychological resource in coping with the condition. These findings underscore the importance of addressing both physical and psychosocial aspects of lipoedema in clinical care. Future research should further explore the role of self-compassion as a protective factor and its potential integration into interventions for patients with lipoedema.

## Data Availability

The data that support the findings of this study are available from the corresponding author upon reasonable request.
