# Supplementary material for: High Self-compassion Despite Elevated Stress and Reduced Quality of Life in Women with Lipoedema
Source: Acta Derm Venereol. 2026 Jun 11;106:0236. doi: 10.2340/actadv.v106.adv-2025-0236 (PMC13254907; doi:10.2340/actadv.v106.adv-2025-0236)
Supplement: Supplementary Material 1. [file ActaDv-106-0236-s0001.pdf]

Appendix S1

Details on psychometric instruments and questionnaires.

Perceived Stress Scale-4

The Perceived Stress Scale-4 (PSS-4) was used to assess perceived stress levels. It consists of four items rated on a 5-point (p) Likert scale, ranging from 0 (never) to 4 p (very often), yielding a total score between 0 and 16 p. Scores from 0 to 8 p are interpreted as indicating normal stress levels, whereas scores from 9 to 16 p suggest elevated stress levels (1). In an English-speaking sample the PSS-4 demonstrated acceptable internal consistency with a Cronbach's alpha of 0.77 (1). The Swedish version has been reported to assess general aspects of stress, rather than specific stressors (2).

Generalised Anxiety Disorder Scale-2

The Generalised Anxiety Disorder Scale-2 (GAD-2) is a brief screening instrument used to assess signs of anxiety. It is a validated short form of the Generalised Anxiety Disorder Scale-7 (GAD-7), developed for use in clinical and research settings (3). The GAD-2 consists of two items rated on a 4-p Likert scale ranging from 0 (not at all) to 3 p (nearly every day), yielding a total score between 0 and 6 p. A score of 0 to 2 p indicates no or mild anxiety symptoms, while a score of 3 p or higher suggests a risk for anxiety disorders and warrants further examination (3). GAD-2 has been validated in multiple studies and has demonstrated strong psychometric properties. While it is more concise, it retains the excellent reliability of the GAD-7, which has been reported to have a Cronbach's alpha of 0.92 and an intraclass correlation of 0.83 (3).

Self-Compassion Scale Short Form

The Self-Compassion Scale Short Form (SCS-SF) was used to assess self-compassion, which is conceptualised as the ability to be understanding and accepting of one's own weaknesses and inadequacies (4). Self-compassion has been associated with psychological well-being and emotional resilience (5). The SCS-SF includes 12 items covering six dimensions of self-compassion: self-kindness, self-judgement, common humanity, isolation, mindfulness, and over-identification. Each item is rated on a 5-p Likert scale ranging from 1 (almost never) to 5 p (almost always), resulting in a total score ranging from 12 to 60 p (4, 5). A mean score between 1.00-2.49 p indicates low self-compassion, 2.50-3.50 p moderate, and 3.51-5.00 p a high self-compassion. SCS-SF is a validated short form of the original Self-Compassion Scale (5) and has demonstrated good internal consistency, with a Cronbach's alpha of 0.85 (4).

Clinical Perfectionism Questionnaire-6

The Clinical Perfectionism Questionnaire-6 (CPQ-6) was used to assess levels of perfectionism, conceptualised as the tendency to strive for achievement and maintain high personal standards (6, 7). The CPQ-6 consists of six items rated on a 4-p Likert scale ranging from 1 (never) to 4 p (always), resulting in a total score between 6 and 24 p. Suggested interpretive categories are as follows: 6-10 p indicate no problems, 11-15 p moderate problems, 16-20 p difficult problems, and 21-24 p severe problems of perfectionism (8, 9). The Swedish version has demonstrated a test-retest correlation of r = 0.62 and an internal consistency of Cronbach's alpha 0.72 (7).

Clance Impostor Phenomenon Scale

The Clance Impostor Phenomenon Scale (CIPS) was used to assess core characteristics of the impostor phenomenon, including self-doubt, fear of failure, and perceived intellectual fraudulence (10, 11). The instrument consists of 20 items rated on a 5-p Likert scale, ranging from 1 (not at all true) to 5 p (very true), yielding a total score between 20-100 p. The scoring system can be interpreted as follows: 20-40 p indicates a low level of impostor characteristics, 41-60 p reflects a moderate level, 61-80 p indicates frequent experiences of the impostor phenomenon, and 81-100 p indicates intense experiences of the impostor phenomenon. In a refinement by Holmes et al., a total score of 62 p or higher has been proposed as a clinically relevant cut-off for identifying individuals with significant impostor feelings (10). The original English version has demonstrated good psychometric properties (10). Although a Swedish translation approved by Dr Clance was used in this study, it has not yet been formally validated (8, 9).

RAND-36

RAND-36 is a validated questionnaire of multiple-choice design containing 36 questions to examine health-related quality of life. The questions are divided into eight subscales with a corresponding physical component summary (PCS) and mental component summary (MCS), each with a range of 0 to 100 points (12). The scores have been validated in both the general population and in various patient groups to assess normative values and the consequences of a condition on physical and mental health (12-14). The prior version of RAND-36, known as SF-36, has been compared to other outcome instruments and found to be a reliable and valid option (15). SF-36 and RAND-36 both have high reliability and good internal consistency, and it has been suggested that they can be used interchangeably when examining health-related quality of life (16).

References

1. Warttig SL, Forshaw MJ, South J, White AK. New, normative, English-sample data for the Short Form Perceived Stress Scale (PSS-4). J Health Psychol 2013; 18: 1617-1628. doi: 10.1177/1359105313508346

2. Rozental A, Forsström D, Johansson M. A psychometric evaluation of the Swedish translation of the Perceived Stress Scale: a Rasch analysis. BMC Psychiatry 2023; 23: 690. doi: 10.1186/s12888-023-05162-4

3. Sapra A, Bhandari P, Sharma S, Chanpura T, Lopp L. Using Generalized Anxiety Disorder-2 (GAD-2) and GAD-7 in a Primary Care Setting. Cureus 2020; 12: e8224. doi: 10.7759/cureus.8224

4. Kotera Y, Sheffield D. Revisiting the Self-compassion Scale-Short Form: Stronger Associations with Self-inadequacy and Resilience. SN Comprehensive Clinical Medicine 2020; 2: 761-769. doi: 10.1007/s42399-020-00309-w

5. Raes F, Pommier E, Neff KD, Van Gucht D. Construction and factorial validation of a short form of the Self-Compassion Scale. Clinical Psychology & Psychotherapy 2011; 18: 250-255. doi: 10.1002/cpp.702

6. Fairburn CG, Cooper Z, Shafran R. Clinical perfectionism questionnaire. PsycTests Database Record 2003; 10.1037/t59141-000. doi: 10.1037/t59141-000

7. Parks A, Van De Leur JC, Strååt M, Elfving F, Andersson G, Carlbring P, et al. A self-report measure of perfectionism: A confirmatory factor analysis of the Swedish version of the Clinical Perfectionism Questionnaire. Clinical Psychology in Europe 2021; 3: e4581. doi: 10.32872/cpe.4581

8. Shayesteh A, Boman J, Nylander E. Impostor phenomenon is a common feature among individuals with primary hyperhidrosis. SAGE Open Medicine 2024; 12. doi: 10.1177/20503121231220828

9. Andersson N, Abdiweli H, Boman J, Nylander E. Self-compassion, perfectionism, impostor phenomenon, stress and anxiety in patients with localized provoked vulvodynia. Journal of Psychosomatic Obstetrics & Gynecology 2023; 44: 2229008. doi: 10.1080/0167482x.2023.2229008

10. Holmes SW, Kertay L, Adamson LB, Holland CL, Clance PR. Measuring the impostor phenomenon: a comparison of Clance's IP Scale and Harvey's I-P Scale. J Pers Assess 1993; 60: 48-59. doi: 10.1207/s15327752jpa6001_3

11. Clance PR. The impostor phenomenon: when success makes you feel like a fake. Clance Impostor Phenomenon Scale (CIPS). Toronto: Bantam Books; 1985

12. Ohlsson-Nevo E, Hiyoshi A, Norén P, Möller M, Karlsson J. The Swedish RAND-36: psychometric characteristics and reference data from the Mid-Swed Health Survey. Journal of Patient-Reported Outcomes 2021; 5: 66. doi: 10.1186/s41687-021-00331-z

13. Hays RD, Sherbourne CD, Mazel RM. The RAND 36-Item Health Survey 1.0. Health Econ 1993; 2: 217-227. doi: 10.1002/hec.4730020305

14. Garratt A, Stavem K. Measurement properties and normative data for the Norwegian SF-36: results from a general population survey. Health and Quality of Life Outcomes 2017; 15: 51. doi: 10.1186/s12955-017-0625-9

15. Angst F, Lehmann S, Aeschlimann A, Sandòr PS, Wagner S. Cross-sectional validity and specificity of comprehensive measurement in lymphedema and lipedema of the lower extremity: a comparison of five outcome instruments. Health and Quality of Life Outcomes 2020; 18: 245. doi: 10.1186/s12955-020-01488-9

16. García-Sánchez E, Santamaría-Peláez M, Benito Figuerola E, Carballo García MJ, Chico Hernando M, García García JM, et al. Comparison of SF-36 and RAND-36 in Cardiovascular Diseases: A Reliability Study. J Clin Med 2024; 13: 6106. doi: 10.3390/jcm13206106
